# Transforming a hospital's organizational culture to promote parent-child relationships and child development

**DOI:** 10.3389/fped.2024.1390770

**Published:** 2024-11-27

**Authors:** Mariel Benjamin, Carrie Quinn, Aliza Pressman, Layla Fattah, Rebecca Parlakian, Ellen Galinsky, Blair Hammond

**Affiliations:** ^1^ Department of Pediatrics, Icahn School of Medicine at Mount Sinai, New York, NY, United States; ^2^ Department of Pediatrics, Zero to Three, Washington D.C., DC, United States; ^3^ Department of Pediatrics, Families and Work Institute, Palisades, NY, United States

**Keywords:** brain development, early learning, messaging campaign, healthcare, positive parenting, culture change, nurturing relationships, organizational culture

## Abstract

**Introduction:**

Early caregiving interactions and experiences profoundly shape a child's brain development. The American Academy of Pediatrics (AAP) recently advocated for a public-health approach to promoting safe, stable, nurturing relationships that is “founded on universal primary preventions”, including consistent messaging on fostering family resilience, nurturing connections, and positive childhood experiences. Hospitals have unique access to families with children ages 0–5 and therefore play a key role in supporting these early experiences. This project sought to shift the organizational culture of maternity and pediatric units at a hospital towards promoting early relationships and child development through a physical messaging campaign paired with staff training. This study examined whether the messaging campaign and staff training shifted staff's self-reported knowledge, attitudes, and behavior.

**Methods:**

Non-physician staff across six pediatric and maternity units in a large urban hospital participated in the intervention. Staff completed surveys before and after message installation and training.

**Results:**

Analysis of 356 pre-intervention surveys and 320 post-intervention surveys showed significant changes in staff's knowledge, attitudes, and behaviors that promote early learning and parent-child relationships. Most staff also reported feeling more empowered in their work (88%) and that the hospital environment had become a friendlier place for parents and families (89%).

**Discussion:**

A messaging and training intervention can create a culture whereby staff support early caregiving and child development in the hospital setting. Further research is needed to understand whether the intervention impacts caregivers and their children.

## Introduction

Early caregiving interactions and experiences profoundly shape a child's brain development (1). Parenting engagement can influence a child's academic success (2), future relationships (3), social-emotional and cognitive growth (4) and even physical health (5). In addition, safe, stable, nurturing relationships (SSNRs) can buffer against the negative impacts of adversity and toxic stress on a child's development and health (6). The American Academy of Pediatrics (AAP) recently advocated for a public-health approach to promoting SSNRs that is founded on universal primary prevention, including consistent messaging on fostering family resilience, nurturing connections, and positive childhood experiences (1).

The hospital and health care system hold unique access to families with children ages 0–5 years – the most critical time for brain development – due to the regular frequency of well-child visits during these early years, and the need for treatment related to the frequent illnesses young children experience (7). In fact, one study found that during the first three years of life, children experience a median 94 days when they have infections (8), For families, it may well feel as though they are “constantly” at the doctor's office during this window in time. A large academic hospital leveraged these regular family interfaces with health care staff in order to deliver messaging and training that supports positive parenting interactions and child development. Existing hospital campaigns designed to provide information about safe sleep and breastfeeding have shown success promoting awareness and behavior change through a dual approach of staff training and public health messaging (9, 10). Harnessing this concept, we sought to apply a similar health communication model regarding the importance of SSNRs. This paper reports an overview of the project and data from the evaluation study.

### The environmental transformation project

This project sought to communicate messages about early parent-child relationships and child development by transforming the organizational culture of maternity and pediatric units at a large, urban, teaching hospital. We employed Schein's theory of organizational culture (11), which has been widely used within healthcare organizations to catalyze cultural change (12–14). This theory posits that organizational culture exists on three levels. The first, the most visible, consists of physical artifacts, or the observable aspects of culture. These reflect the second level, which consists of values and beliefs, at least as articulated by individuals. These, in turn, are informed by basic underlying assumptions, the third level of organizational culture. These are the widely accepted beliefs about human nature and reality that may be unconscious and/or unstated.

Change that is not rooted in culture can often be transient. Schein suggests that every culture has strengths that can be drawn upon and reframed to be supportive of the desired change. In this way, new behaviors and values can be tied to existing assumptions and values, given legitimacy, and made more acceptable and less threatening. Over time, these new behaviors are tried, and if people recognize that they work well to bring about desired results, new assumptions arise to articulate the cultural lessons learned.

The Environmental Transformation Project was designed to change parents' and children's experiences of health care by targeting all three levels of the organizational culture surrounding caregivers and children aged 0–5 at our hospital. Consequently, the project entailed not only transforming the physical environment but also the values and assumptions held by all staff who interact in any way with caregivers of children in that age group. Physicians themselves were targeted in a separate, more intensive medical education curriculum initiative focusing on well child care. The underlying goal was to ensure that the messages being shared with patients through visible artifacts were reinforced through patients' interactions with all types of hospital staff. We now describe how that goal was achieved.

## Methods

### The intervention

**Figure 1:**
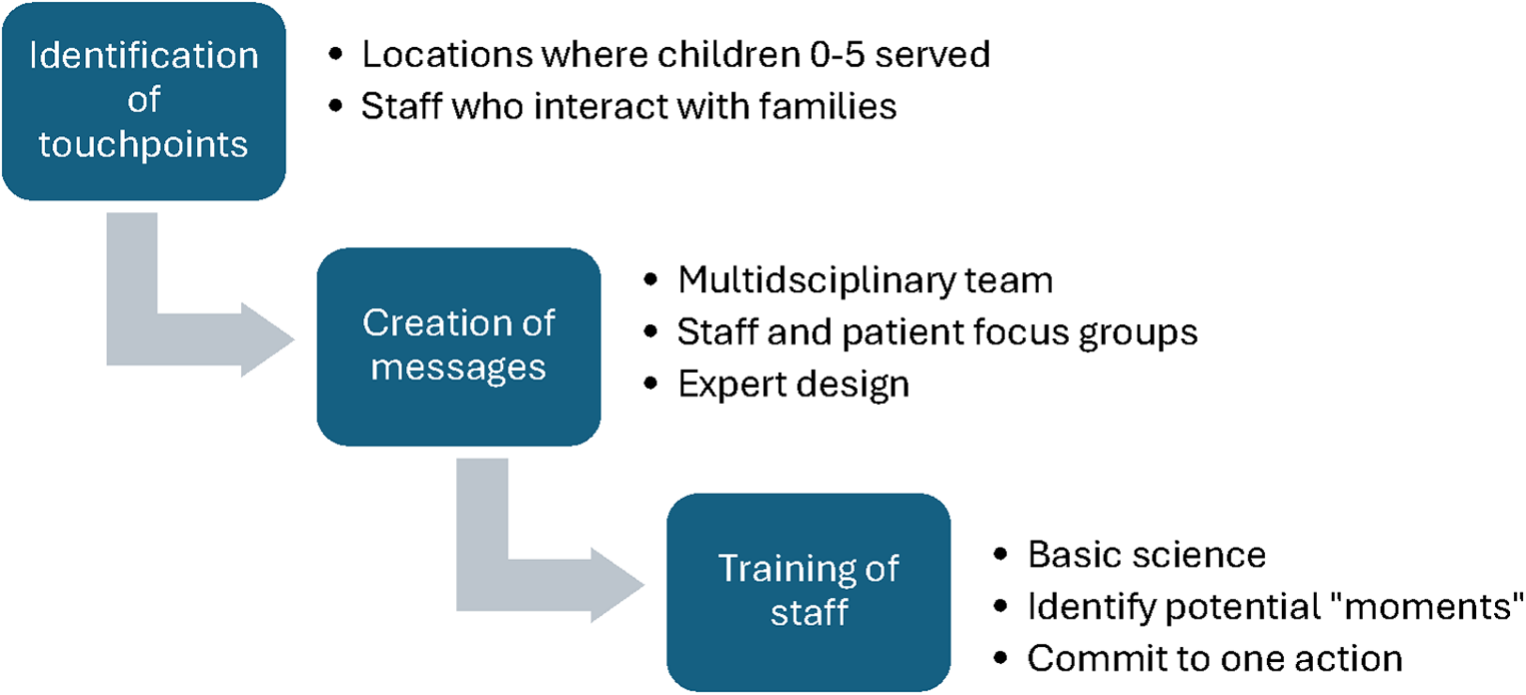
Design of the environmental transformation project.

Figure 1 depicts the three stages of project development. In the first step, a multidisciplinary team identified “touchpoints” throughout six units within the hospital for transformation: the Prenatal Clinic, Labor and Delivery, Postpartum Units, Neonatal Intensive Care, Pediatric Primary Care Clinic, and Pediatric Emergency Department. The Pediatric Inpatient Unit was not included because it does not specifically target young children. This involved, first, identifying all hospital staff who engage with infants and children up to age 5 and their parents. Staff represented food services, housekeeping, transport, and security, in addition to traditional non-physician clinical roles such as nurses, lactation consultants, and medical assistants. Second, the team identified the physical locations, such as elevator banks, front desks, mirrors, and hand sanitizers, which were most visible to families.

In the second step, the team created staff- and family-facing messages in English and Spanish to promote SSNRs and child development. These messages were intended to serve as prompts for interactions between caregivers and children and between staff members and caregivers. Unit staff and families from the hospital's Patient Advisory Committee reviewed the content for relevance and tone. Staff focus groups explored the feasibility and ease of integrating these messages into healthcare interactions, while family focus groups provided feedback on messages' content, inclusivity, and relatedness.

**Figure 2:**
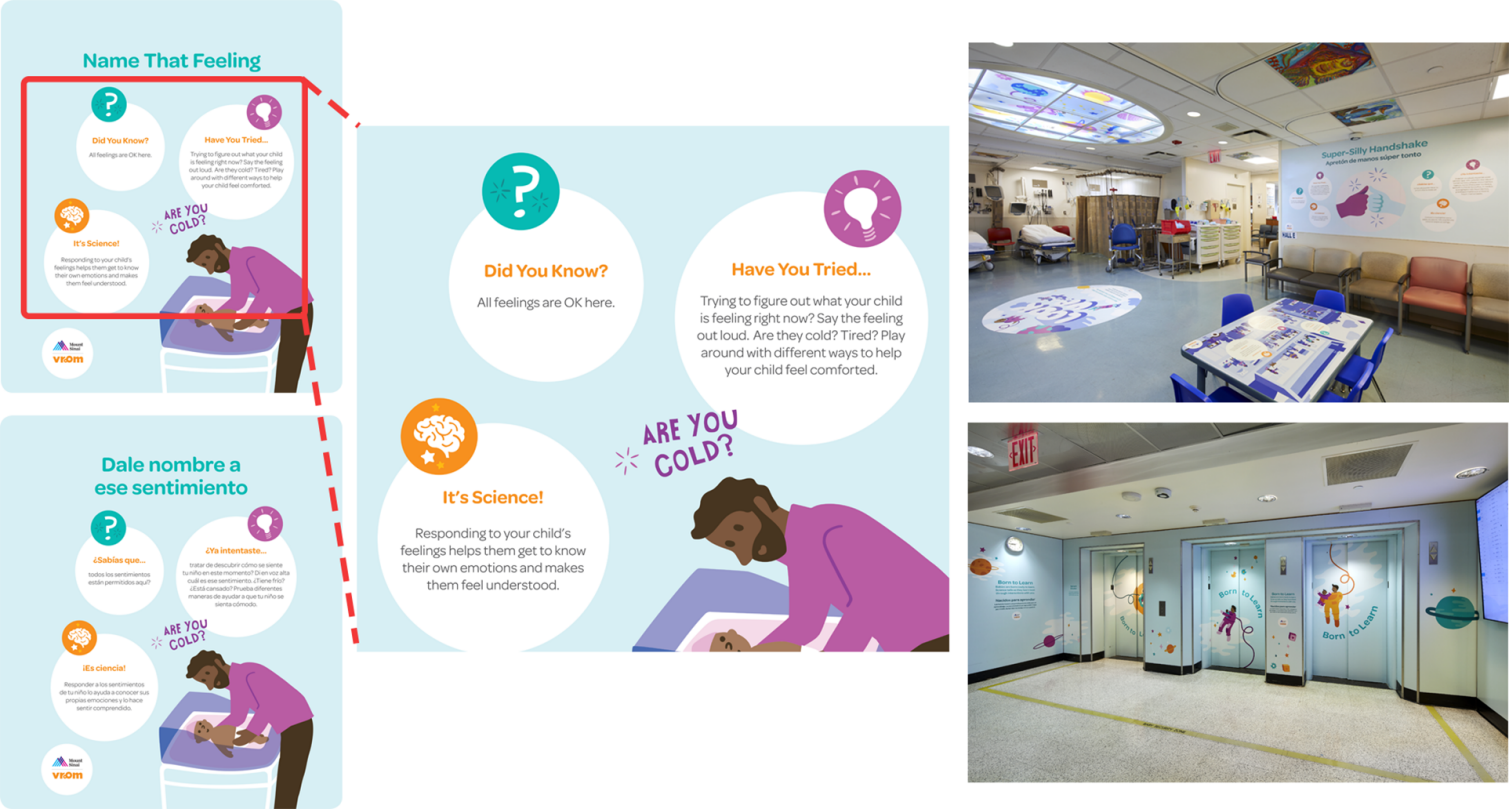
Project images.

An expert design team created over 100 images to support the approved messages. A multidisciplinary panel then reviewed the final illustrated messages. They all shared a similar format. They each begin with “Did you know?” and then offer a piece of information about how children develop, followed by “Have you tried?” and a specific activity to encourage engagement between caregivers and children, followed by “It's science!” and a brief scientific explanation about why the activity is important. Messages focused on themes such as the rapid development of a young child's brain, the importance of everyday interactions with caregivers, and how professionals in all roles can support positive parenting by modeling, discussing, and praising (Figure 2). All messages were framed in a strengths-based fashion (i.e., building on families' existing competencies), which reflected the foundational values of respect and dignity, information-sharing, and participation in family-centered care (15). Whenever possible, messages were tied into the surrounding environment, e.g., meals served to postpartum mothers were set on a paper placemat messaging the importance of mealtimes as opportunities for caregivers to discuss family traditions with children.

The third step of the intervention was the creation and delivery of staff training forums. Shortly after the messages were installed, staff in the six selected units were invited to participate in a 45-minute training designed to teach any staff member, clinical or not, who interacts with caregivers and children how they can use existing opportunities in their workflow to promote SSNRs and early child development. A 10-minute introductory video explained early brain development and described specific parenting behaviors that support healthy child development. Following the video, facilitators encouraged staff to identify the ways they could apply these concepts to their specific role in the hospital. At the conclusion of the session, each participant identified a specific moment in their work (e.g., greeting families, making beds, delivering meals) when they could share messaging from the project, and committed to one action they would take in the future. Locke and Latham's (16) goal setting theory of motivation suggests that the formulation of this specific and clear intention may be linked to enhanced performance in practice. Two weeks after the training, an e-learning module was sent to staff to reinforce the training. In addition, monthly newsletters and frequent unit visits from project staff were also conducted to maintain engagement and awareness.

### The evaluation

We conducted an evaluation to examine whether the environmental messaging and staff training shifted staff's self-reported knowledge, attitudes, and behaviors in the healthcare setting. Data collection first occurred between June 2019 through September 2020. There was a pause from March to August 2020 due to the COVID-19 pandemic, and data collection resumed in September 2020. The authors' Institutional Review Board deemed the study exempt.

#### Participants

Non-physician staff from the six participating hospital units were recruited 1–4 weeks before the messages were installed and the training occurred. Participants represented a wide range of roles, including nursing, medical assistants, social workers, lactation consultants, administration, housekeeping, food services, security, and transport. In each unit, we attempted to recruit 75% of staff for the pre-intervention survey.

We used a modified Delphi approach (17) to develop the survey instrument, which was reviewed by an interdisciplinary panel. Survey questions tapped participants' knowledge of early childhood development, attitudes about staff's role in promoting SSNRs and early brain development, their frequency of behaviors that promote SSNRs and early brain development, and perceived barriers to promoting SSNRs and early brain development.

Approximately 1–3 months after installing the messages and staff trainings, we administered a post-intervention survey. Almost all of the participants in the post-intervention survey attended a training. Furthermore, participation in the post-intervention survey was not conditioned on participation in the pre-intervention survey. All data collection occurred anonymously, making it impossible to link the respondents across the two surveys.

#### Measures

Both the pre- and post-intervention survey asked respondents to endorse five knowledge statements (e.g., “The brain grows fastest during the first five years of life”) and two attitude statements (e.g., “Hospital staff can promote early brain development during typical interactions”) using a 5-point Likert scale (1 = strongly disagree, 5 = strongly agree). For the purpose of analysis, we selected the proportion who selected strongly agree. Respondents were also asked which of five strategies for early learning (e.g., responding to the child's sounds, actions or words) they discussed, modeled, or praised in interactions with caregivers, and which of five messages (e.g., “Positive relationships with caring adults are critical for early brain development”) they discussed with caregivers using a yes/no checklist.

Three barriers to implementing the lessons from the training – lack of confidence, lack of buy-in (i.e., the belief that it was not part of their job), and lack of feasibility – were also assessed. Respondents were asked about their confidence, their buy-in and the feasibility of three behaviors: praising caregivers for what they are already doing, demonstrating positive adult-child interactions, and sharing information or messages about early learning and brain development. Responses were provided on a 5-point scale (1 = not sure, 5 = very much so); for analysis, we selected the proportion answering very much so. The post-intervention survey also gathered feedback on the training experience. The only information collected about respondents was their role and unit.

#### Analytic plan

Data were analyzed using IBM SPSS Statistics. Study respondents were characterized using descriptive statistics, and *t*-tests for independent samples were used to test for pre- and post-intervention differences on individual survey items. The *p-*value was set to 0.05.

## Results

### Staff knowledge, attitudes, and behaviors

The pre-intervention survey was completed by 356 staff across the six units, and the post-intervention survey was completed by 320 staff across the six units. There were no differences in terms of role and unit between the pre- and post-intervention survey respondents, with the exception of the Labor and Deliver unit, where the proportion of respondents who were nurses was higher in the post-intervention sample (see Appendix A for roles of participants).

Results showed that compared to before the intervention, after the intervention, staff scored statistically significantly higher on all attitude questions, and on four out of the five knowledge questions (Table 1). Staff were also statistically significantly more likely after the intervention than before to engage in all behaviors that promote early learning during interactions with caregivers (Table 2). They were also statistically significantly more likely to communicate all messages that promote early learning to caregivers.

**Table 1:** Knowledge and attitudes, Pre- and post-intervention.

	Pre-intervention (*n* = 356) % strongly agree	Post-intervention (*n* = 319) % strongly agree
Knowledge		
Positive relationships with caring adults are critical for early brain development	87	91
Parents can promote early brain development as part of daily routines	85	91*
It is important that parents talk to their children for early brain development – even before their children can talk	85	91*
Children start learning from the moment they are born	83	88*
The brain grows fastest during the first five years of life	76	88*
Attitudes		
At this hospital, we all have a role in encouraging children's early learning and brain development	66	84**
Hospital staff can promote early brain development during typical interactions	58	80**

* *p* < .05.

** *p* < .01.

**Table 2:** Behaviors, pre- and post-intervention.

	Pre-intervention (*n* = 356) % yes	Post-intervention (*n* = 319) % yes
Behaviors with caregivers/children		
Responding to the child's sounds, actions or words	83	91**
Making eye contact and looking into the child's eyes	82	92**
Paying attention to what the child is showing interest in	78	88**
Having back and forth interactions with the child (taking turns)	70	82**
Talking out loud about the things you are seeing, hearing and doing	70	84**
Asking the child open ended questions, like “what do you think about that?”	62	72*
Messages communicated with caregivers		
Positive relationships with caring adults are critical for early brain development	74	83**
Parents can promote early brain development as part of daily routines	71	80*
It is important that parents talk to their children for early brain development – even before their children can talk	71	83**
Children start learning from the moment they are born	74	88*
The brain grows fastest during the first five years of life	62	73**

* *p* < .05.

** *p* < .01.

Further, after the intervention, staff were more confident than before in their ability to engage in behaviors that promote SSNRs and early child development; more likely to believe that it was part of their role to routinely do so; and more likely to consider doing so feasible (Table 3). This was true of all three behaviors: praising caregivers, demonstrating positive adult-child interactions, and sharing information or messages. Further, 95% of staff reported that they were successful in making the change in practice they had resolved to make at the end of their training (not shown).

**Table 3:** Potential barriers to implementation, pre- and post-intervention.

	Pre-intervention (*n* = 356) % very much so	Post-intervention (*n* = 319) % very much so
How confident do you feel in your ability to routinely…		
Praise caregivers for what they are already doing	64	77**
Demonstrate positive adult-child interactions	59	73**
Share specific information or messages about early learning and brain development	49	63**
To what extent do you believe it is part of your role to routinely…		
Praise caregivers for what they are already doing	70	81**
Demonstrate positive adult-child interactions	67	78**
Share specific information or messages about early learning and brain development	55	69**
In your setting, how feasible/realistic do you believe it is for someone in your role to routinely…		
Praise caregivers for what they are already doing	58	71**
Demonstrate positive adult-child interactions	50	68**
Share specific information or messages about early learning and brain development	42	62**

** *p* < .01.

### Post-Intervention perception of the hospital environment

We asked participants in the post-intervention survey to reflect on the impact of the project both on their perception of their role and on the hospital environment. Most staff (89%) agreed or strongly agreed that as a result of the project, they were more patient- and family-centered in their interactions (not shown); most (88%) agreed or strongly agreed that they felt more empowered in their work; and most (89%) agreed or strongly agreed that the hospital environment had become a friendlier place for parents and families.

## Discussion

This study evaluated a project that aimed to empower non-physician staff through a environmental messaging and training campaign focused on enhancing communications with families about early child development and positive parenting behaviors. Using visible artifacts and developing shared organizational values and beliefs through staff training led to significant improvement in staff's knowledge, attitudes, and behaviors. Prior to the intervention, staff reported high baseline knowledge around early child development, but did not report consistently communicating this information with caregivers as they did after the intervention. In addition, following the intervention, staff's beliefs and attitudes shifted so that the majority viewed the routine discussion of positive caregiver behaviors with families as one of their responsibilities. In fact, after the intervention, 95% of staff reported behavior change that promoted child development. This transfer from knowledge to practice, and from not viewing this to viewing this as part of their professional role, suggests that the project was successful in improving the hospital culture. The fact that 88% of respondents felt that the intervention made them feel more empowered also suggests that it may have enhanced the overall workplace environment.

Other hospital-based projects have successfully combined public-facing messages with in-staff training in the service of health promotion [e.g., Pineda et al. (18)], but such projects typically include clinical staff only. Lucarelli et al. (19) conducted training for front-line staff to familiarize them with, and prepare them for treating, children with autism spectrum disorder; these included front-desk staff but did not routinely involve staff from services such as housekeeping and food service. Yet an organization's culture is both internalized and expressed by staff in all roles, and any attempt to change that culture by transforming the physical environment alone is likely to be insufficient without concomitant attention to the values and assumptions held by members of that organization (12, 14).

Our aim was to provide both staff and caregivers with one consistent message: that young children's interactions with caregivers are of vital importance for child development. The evaluation results suggest that a highly targeted but relatively low-intensity intervention may be an effective way of delivering this message while also improving family-centered care. Existing research indicates that family-centered care models, policies, and programs designed to “help sustain” the caregiver role may “optimize care provision in the home” (20 p. 2). Establishing a clearer pathway of how professional interactions can contribute to positive family functioning may spotlight an important opportunity to mitigate risk factors and increase family resilience.

## Limitations

This study has several limitations to consider. First, to some extent, respondents to the pre-intervention survey may not overlap with the respondents to the post-intervention survey. Second, those surveys relied on self-report, which may have been influenced by social desirability. Replication of this project with an observational study design would be valuable. Third, the post-intervention surveys were gathered within one month of the training, and lasting effects were not measured. Fourth, the COVID-19 pandemic disrupted data collection and had long-term implications for hospital flow and usage of unit space. The stress, strain, and hardship endured by patients and staff during the pandemic necessitated both structural and support changes that may have influenced the project's impact. Finally, it is unknown whether the intervention actually changed caregivers’ knowledge, attitudes, and behaviors, and if so, whether those changes resulted in improved developmental outcomes for children.

## Conclusion

This evaluation study is the first step towards understanding the effectiveness of a large-scale environmental messaging campaign aimed at engaging healthcare staff in strengthening SSNRs and promoting early child development. Evidence from other messaging and training campaigns (9, 10, 14, 18, 19), paired with the results from this intervention, suggest that shifting culture in healthcare settings is not only possible, but also worthwhile.

We believe this pairing of environmental prompts and professional guidance may serve as a health communication model for sparking change in the service of family-centered care practices and, in the case of our study, an increased sense of staff satisfaction and empowerment. Improvement in family-centered care and staff satisfaction are also key quality metrics for healthcare institutions, and interventions that can influence those outcomes warrant further investigation.

While designed for national adoption, long term assessment of this intervention is required to test whether this is a sustainable model to promote rich staff communication with families and positive parenting interactions and healthy development. Valuable avenues of future study might build on these initial findings to deepen the field's collective understanding of how visual assets, actionable messaging, and staff training can be combined and directed to different types of staff, and different populations of families, to achieve cultural change while improving family-centered care and supporting staff empowerment.

## Data Availability

The raw data supporting the conclusions of this article will be made available by the authors, without undue reservation.

## Appendix A Participant roles and numbers.

**Table:**

Role	Pre-intervention	Post-intervention
	*N* = 361	*N* = 328
Nurse	126	145
Business associate(BA)	47	32
Technicians	31	30
Social Worker	27	14
Patient care advocate/Associate	23	19
Registrars	14	12
Therapist/Specialist	10	9
Nursing assistant	9	7
Medical assistant/Associate	8	16
Nurse practitioner	7	3
Administrative staff	7	5
Midwife	4	2
Lactation consultant	4	4
Housekeeping	4	0
Food services	3	1
Clinical/Care coordinator	3	3
Photographer	2	3
Patient transporter	2	0
Guest/Patient services management	1	8
Clinical nurse manager	1	4
Other	15	9
Missing	13	2
